# Outcomes of transforaminal epidural injection of amniotic membrane/umbilical cord particulate for lumbar radiculopathy: a case series

**DOI:** 10.3389/fpain.2024.1322848

**Published:** 2024-01-22

**Authors:** Mark Miedema, Angela Anderson

**Affiliations:** Department of Orthopaedics, Ozark Orthopaedics, Fayetteville, AR, United States

**Keywords:** amniotic, back pain, chronic pain, epidural, lumbar radiculopathy, pain, radiculopathy, umbilical cord

## Abstract

**Background:**

Radiculopathy can be a debilitating condition. Amniotic membrane/umbilical cord (AM/UC) particulate is a relatively new injectable treatment modality. Herein we report the outcomes of epidural injection of AM/UC particulate in managing lumbar radiculopathy.

**Methods:**

Consecutive patients with lumbar radiculopathy who received epidural injection of AM/UC particulate for lumbar radiculopathy were included. Primary outcome was change in pain as measured by the 11-point numerical rating scale. Safety was assessed by AM/UC- and procedure-related complications. Paired t-tests were used to determine statistical significance.

**Results:**

A total of 12 patients with a mean age of 56.7 ± 21.0 years were included in the analysis. The patients were previously treated with physical therapy (91.7%), chiropractic corrective measures (16.7%), epidural steroid injection (83.3%), and radiofrequency ablation (8.3%). Two patients (16.7%) were taking opioids for chronic pain syndrome. After AM/UC injection, the average pain score decreased from 6.6 ± 1.5 to 5.2 ± 1.9 at 1–3 months, 2.0 ± 1.4 at 6 months, and 2.9 ± 1.4 at last mean follow-up of 21.3 ± 11.1 months (*p* < 0.001). No patients required subsequent treatment or surgery. There were also no complications.

**Conclusion:**

This case series supports the preliminary safety and shows potential benefit of epidural AM/UC particulate injection in this cohort of patients with lumbar radiculopathy pain.

## Introduction

Radiculopathy is a diffuse spinal condition characterized by mechanical compression or damage to one or more spinal nerve roots. The most common causes of radiculopathy include intervertebral herniated disc, spondylosis, spondylolisthesis, and ligamentum flavum hypertrophy which result in nerve root compression. Symptoms can be multitude including back and leg pain with or without extremity paresthesia, loss of sensation, or loss of motor function depending on the severity of compression, and damage to the nerve roots. These symptoms are often debilitating, and more than half of these patients report a disturbance in their activities of daily life. Radiculopathy is also relatively common with an annual incidence of 83–1,200 per 100,000 persons (1).

Conventional therapy generally includes physical therapy, activity modification, and pain medication. However, these treatment benefits can be relatively short-lived and many patients continue to have severe pain (2). For cases that don't respond to conservative treatment for over six weeks, epidural steroid injections are often employed to reduce inflammation and subsequently relieve pain. These injections generally provide a moderate, but clinically significant reduction in pain for up to three months post-injection (3). However, the outcomes of these injections are less predictable and less beneficial in patients with chronic radiculopathy. In one study, ∼10% of patients eventually required surgery for severe pain despite receiving multiple epidural steroid injections for their pain (4). Hence, there remains an unmet clinical need for managing pain associated with radiculopathy in patients who wish to avoid surgery.

Recently, the use of biological therapies to treat chronic, painful conditions has become increasingly popular. In particular, over the past ten years, amniotic membrane and umbilical cord (AM/UC) particulate has demonstrated safety and effectiveness in treatment of a wide range of musculoskeletal indications including knee osteoarthritis (5), wrist osteoarthritis (6), spine facet osteoarthritis (7), and discogenic pain (8). The rationale of using AM/UC in musculoskeletal indications manifesting pain is based on its anti-inflammatory, anti-scarring, and pro-regenerative properties which are thought to reduce inflammation and promote restorative healing in the local environment (9–12). Such effects have not only provided a rapid pain relief, but also longstanding relief for 6 to 12 months after a single injection. Due to these benefits, AM/UC particulate injection may also provide symptomatic pain relief in patients with lumbar radiculopathy. Herein we assessed lumbar radiculopathy patients treated with AM/UC epidural injection to determine the safety and effectiveness in real world evidence from routine clinical practice.

## Materials and methods

Following review and exemption by Sterling Institutional Review Board (IRB), a retrospective review was performed on consecutive patients receiving epidural injection of AM/UC particulate for lumbar radiculopathy by a single physician between May 1, 2019, and January 1, 2022, at the author's practice. The study was conducted in accordance with the Declaration of Helsinki. In accordance with the Health Insurance Portability and Accountability Act (HIPAA), only minimum necessary data were extracted from electronic medical records, which included age, gender, diagnosis, comorbidities, prior and concomitant medications, complications and pain score. Patients were eligible for inclusion if they received epidural injection of particulate AM/UC for lumbar radiculopathy and provided a pain score before and after injection. Patients were excluded if there was no pre- or post-procedure pain scores available in the medical records.

All patients were treated with AM/UC particulate in a similar manner via transforaminal approach. Briefly, the patient is placed in prone position with a pillow under the lower abdomen to reduce normal lumbar lordosis. IV sedation was performed for analgesia during the procedure. At the designated spinal level, skin and subcutaneous tissue were anesthetized with 1% lidocaine. A spinal 22 gauge needle (5″ Quincke) was then guided into superior-anterior neuroforamen lateral to mid-pedicular line using intermittent fluoroscopy. Bony landmarks of neuroforamen were identified to guide the oblique approach. Once the needle was in position, aspiration was performed to confirm no blood or cerebrospinal fluid. An extension tube was attached to the spinal needle and 2cc Omnipaque 240 (nonionic contrast agent) was injected under real time fluoroscopy. Correct needle placement was confirmed by production of epidurogram and radiculogram without concurrent vascular update. A syringe with AM/UC (50 mg or 100 mg) pre-mixed with 4cc of 1% lidocaine was then connected to the needle and injected into the epidural space. The needle was then slowly removed and the patient was moved to the recovery unit in the supine position.

The AM/UC particulate (Clarix® Flo; BioTissue, Miami, FL) has been commercially available since 2013 and is derived from donated human placental tissue following healthy, live, caesarian section, full-term births. The donor of the placental tissue is screened to ensure the absence of infectious, malignant, neurological, and auto-immune diseases. During the processing of the AM/UC tissue, the tissue is cleansed, lyophilized via freeze drying, micronized into particulates, and sterilized via gamma irradiation. According to the manufacturer, the product preserves the natural biological characteristics innate to fresh AM/UC tissue but not any living cells (12).

After injection, the patients' vital signs and pulse oximetry were continuously checked during recovery. The patients were discharged from conscious sedation to the care of their caretaker when they were stable without drowsiness or pain (usually 10–15 min). For any post-procedure pain relief, they were advised to use acetaminophen 325 mg every 6 h as needed and to avoid non-steroidal anti-inflammatories for one week. Patients were instructed to rest the day of the procedure and resume normal activities the following day. Additionally, they were advised to avoid strenuous exercise for at least one week. The patients were instructed to seek immediate medical attention for shortness of breath, chest pain, fever, chills, increased pain, weakness, sensory or motor changes, or changes in bowel or bladder function.

The outcomes evaluated were pain related to radiculopathy and any complications attributable to the injection (e.g., nausea, headache, dizziness, redness, neural injury, bleeding, infection, bruising, or epidural abscess). For pain, the numerical rating scale was used in which patients reported the average severity of their pain over the last week on a 11-point scale, ranging from “no pain” at 0 to “worst imaginable pain” at 10. The outcomes were retrieved from the electronic medical records and were analyzed based on follow-up exams on each visit the patient made to the clinic. The proportion of patients who needed subsequent treatment including surgery was also documented.

### Statistical analysis

All statistical analyses were performed in R version 3.6.2. Descriptive statistics were used to report the demographic and clinical characteristics. Categorical variables were described with percentages and frequencies, while continuous variables were described with mean ± standard deviation or median (range). The 95% confidence intervals (95% CIs) were provided for pain scores. Paired t-tests were used to determine whether there is a statistically significant improvement of pain at follow-ups compared to baseline. A *P*-value of <0.05 was considered significant.

## Results

During the specified time period, there were a total of 12 patients that received epidural injection of AM/UC particulate for lumbar radiculopathy. All patients (100%) met the eligibility criteria and were included in the analysis. The population included 6 males and 6 females with a mean age of 56.7 ± 21.0 years. Comorbidities include hypertension (41.7%), depression (16.7%), high cholesterol (33.3%), diabetes (16.7%), thyroid conditions (16.7%), asthma (16.7%), and gastric reflux (8.3%). Additionally, three patients were smokers, two of whom had been smoking for over 10 years. Prior to AM/UC particulate injection, the mean patient pain score was 6.6 ± 1.5 despite previous treatment with physical therapy (91.7%), chiropractic corrective measures (16.7%), epidural steroid injection (83.3%), and radiofrequency ablation (8.3%). One patient had prior discectomy at same level of radiculopathy. Two patients (16.7%) were taking opioids for chronic pain syndrome.

The AM/UC injection was performed uneventfully in all cases. Patients received transforaminal injection of 50 mg (58.3%) or 100 mg AM/UC (41.7%) at one (41.7%) or two (58.3%) levels (Table 1). Two of the 5 patients receiving 100 mg AM/UC had the particulate mixture injected across two injection sites (epidural and intradiscal).

**Table 1:** Characteristics of population.

#	Age	Gender	Injection location(s)	AM/UC amount (mg)	Pre-pain score	Pain score at last follow-up	Follow-up duration (months)
1	37	F	L5-S1, S1	50	8	2	20
2	89	F	L5-S1	50	5	5	19
3	48	F	L4-5^a^, L5-S1	50	7	3	18
4	18	M	L5-S1^a^, L5-S1	100	5	2	14
5	50	M	L4-5, L5-S1	100	7	1	13
6	53	F	L4-5, L5-S1	50	7	6	6
7	35	F	L5-S1	50	7	4	4
8	80	M	L4-5, L5-S1	50	8	2	38
9	67	M	L4-5, L5-S1	50	7	2	36
10	59	M	L5-S1	100	3	3	30
11	62	F	L5-S1	100	7	3	29
12	82	M	L5-S1	100	8	2	28

M, male; F, female.

^a^ Intradiscal.

After AM/UC injection, the average pain score decreased to 5.2 ± 1.9 at 1–3 months (3.68–6.72 CI; *n* = 6; *p* = 0.110), 2.0 ± 1.4 at 6 months (0.06–3.94 CI; *n* = 2; *p* = 0.323), and 2.9 ± 1.4 at last mean follow-up of 21.3 ± 11.1 months (2.11–3.69 CI; *n* = 12; *p* < 0.001). No patients required subsequent treatment or surgery for their radiculopathy and there were no complications or adverse events attributed to injection of AM/UC. There was no change in opioid use when comparing medication usage before injection to medication usage after injection (*p* > 0.05). Of note, the one patient that previously suffered from failed back surgery syndrome had their pain level decrease from 7 to 4 after receiving AM/UC injection.

## Discussion

Transforaminal epidural injections are a mainstay treatment option for many pain specialists, including anesthesiologists, physiatrists, and radiologists. Generally, these injections are performed with steroids such as methylprednisolone, hydrocortisone, triamcinolone, betamethasone, and dexamethasone (13, 14), though they are not FDA approved specifically for such use in the epidural space. Epidural steroid injections generally provide short-term relief for up to three months though literature suggests longer lasting effects in some cases. A recent meta-analysis showed 41.7% of patients receiving epidural steroid plus local anesthetic injection had significant improvement in pain with an average total relief for 31.7 ± 13.2 weeks per year (15). However those patients received multiple injections with an average 3.68 ± 1.17 injection per year, hence there can be a relatively short-lived benefit with steroid adminisitration. Furthermore, the outcomes of these injections may be less favorable in patients with chronic disease. Manchikanti et al. compared transforaminal epidural injection of lidocaine and saline with lidocaine and betamethasone and found the use of adjunctive steroids did not help improve outcomes in patients with chronic lumbar disc herniation (16). Steroid injections also carry potential complications of hyperadrenocorticism, Cushing syndrome, osteoporosis, avascular necrosis, myopathy, epidural lipomatosis, fluid retention, and hyperglycemia (17, 18). Hence, there remains an unmet clinical need for managing pain associated with radiculopathy.

Using the similar transforminal epidural technique that one would use with steroids, we evaluated the epidural injection of AM/UC injection. Patients were shown to have a significant reduction in pain that lasted 21.3 ± 11.1 months after a single AM/UC injection, despite all patients failing previous conservative treatments including physical therapy (91.7%) and/or epidural steroid injection (83.3%). No patient required spinal surgery during the follow-up and no adverse events were observed, despite the known risks of transforaminal epidural injection with a particulate (19). Additionally, the mean improvement in pain at 6-months and last follow-up (3.2 and 2.3, respectively) is greater than the minimal clinically important change (MCIC) for chronic low back pain according to literature (2.0) (20, 21). Furthermore, these results are consistent with other studies in which AM/UC showed lasting benefit in conditions such as knee osteoarthritis, facet joint osteoarthritis, rotator cuff tear, discogenic pain, and peripheral neuropathy (6, 7, 22–27). In particular in those patients with facet joint syndrome, prior studies have shown AM/UC intra- or peri-articular injection improved symptoms 59%–95% at 6 months, similar to the 62% change seen in our case series. The benefits of AM/UC injections in musculoskeletal pathologies may be based on its reported therapeutic actions in reducing inflammation, inhibiting scar tissue formation, and supporting stem cell function. AM/UC contains growth factors, cytokines and ECM components including HC-HA/PTX3, which has been shown to be the major tissue component uniquely present in AM/UC that is also vital to its ability to promote apoptosis of pro-inflammatory cells (28) and suppress fibrosis (29). Unlike steroids, HC-HA/PTX3 acts only on activated pro-inflammatory cells (as opposed to resting, inactivated immune cells) to promote their cell death and acts on neutrophils, macrophages, and lymphocytes for a broad spectrum effect (30, 31). Collectively, these actions create a regenerative response which may be translated to lasting benefits (30, 31), in particular in those patients that have failed prior therapies and/or where other therapies are contraindicated.

Candidates for AM/UC may be those patients that have failed prior therapies and/or where other therapies are contraindicated. The benefits of AM/UC may also extend to patients with failed back surgery syndrome, of which one of our patients suffered and noted benefit after receiving AM/UC injection. Studies have conservatively estimated that approximately 20%–40% of patients undergoing surgery may experience post-laminectomy syndrome (32). Continuing pressure on the nerve root after lumbar surgery is one of the common causes of persistent symptoms. Compared to patients with other chronic pain syndromes, these patients have been shown to exhibit lower quality of life scores and higher amounts of pain, unemployment, opioid use, and disability (33). For these patients, reoperation may be an option for patients with clearly identified pathologies that may be relieved with surgery. However, reoperation generally correlates with inferior outcomes and higher morbidity. (34). Hence having an alternative such as AM/UC injection may be advantageous in this patient cohort.

Despite the preliminary beneficial findings of our study, there are limitations to note. The retrospective design was reliant on data collected from patients that returned to the office for follow-up. Of note, all patients that received AM/UC particulate for lumbar radiculopathy during the inclusion criteria returned for follow-up and had their pain score recorded. Furthermore, all patients received epidural of AM/UC particulate that was reconstituted in 1% lidocaine, which may have an analgesic effect in itself. Lidocaine is recommended in epidural mixtures to enable a rapid neurological evaluation and has been shown to provide benefit in 50%–80% of patients for up to two years (35, 36). However, 10 of the 12 patients in this case series had prior steroid plus lidocaine epidural injection that failed to resolve their symptoms, suggesting the benefit observed in our study was most likely attributable to AM/UC particulate. Furthermore, the sample size was relatively small, the use of opioid did not significantly differ before and after AM/UC particulate, and a retrospective design is not optimal for investigating the efficacy and safety of an intervention. Future randomized controlled studies are warranted to further assess safety and efficacy of AM/UC injection in treatment of radiculopathy associated pain, as well as its health economic evaluation.

## Data Availability

The raw data supporting the conclusions of this article will be made available by the authors, without undue reservation.
